# Supplementary material for: Long-term follow-up of patients discontinuing bulevirtide treatment upon long-term HDV-RNA suppression
Source: JHEP Rep. 2023 Apr 7;5(8):100751. doi: 10.1016/j.jhepr.2023.100751 (PMC10285645; doi:10.1016/j.jhepr.2023.100751)
Supplement: Multimedia component 1 [file mmc1.pdf]

Supplementary material

Long-term follow up of patients discontinuing bulevirtide treatment upon long-term HDV-RNA suppression

Mathias Jachs, Marlene Panzer, Lukas Hartl, Michael Schwarz, Lorenz Balcar, Jeremy V. Camp, Petra Munda, Mattias Mandorfer, Michael Trauner, Stephan W. Aberle, Heinz Zoller, Thomas Reiberger, Peter Ferenci

Table of contents

Fig. S1 Page 2
Table S1 Page 3
References Page 4

Supplementary Figure legends

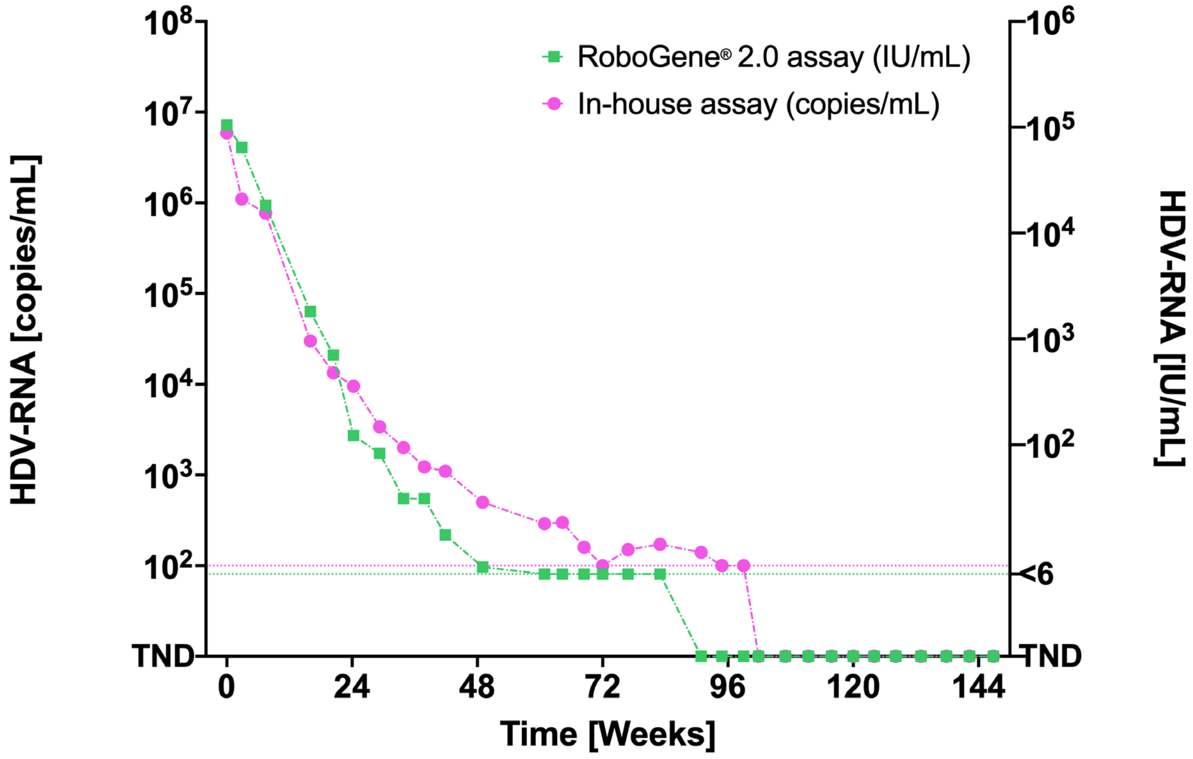

Fig. S1. Head-to-head comparison of the diagnostic performance of the HDV-RNA assay developed in-house according to Le Gal et al. [1] (lower limit of linear quantification: 100 copies/mL) used in six out of seven included patients, and the RoboGene ® 2.0 assay (lower limit of linear quantification: 6 IU/mL), over the bulevirtide (BLV) treatment course of patient #1. The assessment applying the RoboGene ® 2.0 assay was conducted externally [2].

Table S1

Patient No.	Treatment	qHBsAg (log$_{10}$ IU/mL)		
		Treatment-naïve	BLV discontinuation	Last Follow-Up
**P1**	BLV	4.03	3.91	3.79
**P2**	BLV	3.22	3.17	3.06
**P3**	BLV	3.56	3.88	3.79
**P4**	BLV + PEG-IFN	4.37	4.36	4.09
**P5**	BLV + PEG-IFN	4.05	2.93	2.86
**P6**	BLV	3.09	3.09	2.88
**P7**	BLV	3.08	2.94	2.91

Table S1: Individual quantitative HBsAg (qHBsAg) levels at treatment initiation, BLV (± PEG-IFN) discontinuation, and at the last recorded follow-up visit.

References

[1] Le Gal F, Gordien E, Affolabi D, Hanslik T, Alloui C, Dény P, et al. Quantification of hepatitis delta virus RNA in serum by consensus real-time PCR indicates different patterns of virological response to interferon therapy in chronically infected patients. J Clin Microbiol 2005;43:2363-2369.

[2] Loglio A, Ferenci P, Uceda Renteria SC, Tham CYL, Scholtes C, Holzmann H, et al. Safety and effectiveness of up to 3 years' bulevirtide monotherapy in patients with HDV-related cirrhosis. J Hepatol 2022;76:464-469.
