# Supplementary material for: Long-term follow-up of patients discontinuing bulevirtide treatment upon long-term HDV-RNA suppression
Source: JHEP Rep. 2023 Apr 7;5(8):100751. doi: 10.1016/j.jhepr.2023.100751 (PMC10285645; doi:10.1016/j.jhepr.2023.100751)
Supplement: Multimedia component 3 [file mmc3.pdf]

Long-term follow-up of patients discontinuing bulevirtide treatment upon long-term HDV-RNA suppression

Mathias Jachs,[1,2,*] Marlene Panzer,[3] Lukas Hartl,[1,2] Michael Schwarz,[1,2] Lorenz Balcar,[1,2] Jeremy V. Camp,[4] Petra Munda,[1,2] Mattias Mandorfer,[1,2] Michael Trauner,[1,2] Stephan W. Aberle,[4] Heinz Zoller,[3] Thomas Reiberger,[1,2,†,*] Peter Ferenci[1,†,‡]

[1]Division of Gastroenterology and Hepatology, Department of Internal Medicine III, Medical University of Vienna, Vienna, Austria; [2]Rare Liver Disease (RALID) Center of the European Reference Network for Rare Hepatological Diseases (ERN RARE-LIVER), Medical University Vienna, Vienna, Austria; [3]Department of Medicine I and Christian Doppler Laboratory on Iron and Phosphate Biology, Medical University of Innsbruck, Innsbruck, Austria; [4]Center for Virology, Medical University of Vienna, Vienna, Austria

JHEP Reports **2023.** https://doi.org/10.1016/j.jhepr.2023.100751

Background & Aims: Bulevirtide (BLV) is a novel antiviral drug licensed for the treatment of chronic hepatitis D. Data on the safety and efficacy of stopping BLV therapy upon long-term HDV-RNA suppression are scarce.
Methods: A total of seven patients (age, 31–68 years, four with cirrhosis) included in a prospective Austrian HDV registry discontinued BLV treatment (duration, 46–141 weeks) upon long-term HDV suppression (HDV-RNA negativity, 12–69 weeks). Pegylated interferon-α2a was used in combination with BLV in two patients. HDV-RNA, alanine aminotransferase, and quantitative HBsAg levels were closely monitored during treatment-free follow-up.
Results: The seven patients were followed up for 14 to 112 weeks. Six patients completed ≥24 weeks of follow-up. HDV-RNA became detectable again in three patients within 24 weeks, whereas one additional patient showed an HDV-RNA relapse after almost 1 year. All patients who relapsed at any point had undergone BLV monotherapy. Meanwhile, HDV-RNA remained undetectable in two patients who were treated with BLV + pegylated interferon-α2a. Only one patient showed significant alanine aminotransferase increases within 24 weeks of follow-up. BLV was reintroduced in three patients after 13–62 BLV-free weeks and was well tolerated, and all patients achieved virologic response again.
Conclusions: BLV discontinuation upon long-term HDV-RNA suppression seems safe. Retreatment with BLV was effective in case of virologic relapse. These findings are within a limited number of patients, and future studies are needed to define stopping rules and further investigate the safety of stopping BLV.
Impact and Implications: Limited data exist on stopping bulevirtide (BLV) treatment in patients who achieve long-term HDV-RNA suppression. In a small cohort of seven Austrian patients discontinuing BLV therapy, HDV-RNA relapses were observed in four patients during long-term follow-up, whereas significant alanine aminotransferase increases were recorded in only one. Retreatment with BLV was effective in relapsers. The safety and efficacy of stopping BLV needs to be further studied in larger cohorts.
© 2023 The Authors. Published by Elsevier B.V. on behalf of European Association for the Study of the Liver (EASL). This is an open access article under the CC BY license (http://creativecommons.org/licenses/by/4.0/).

Introduction

Chronic hepatitis D (CHD) is a dangerous form of chronic viral hepatitis and has been associated with a more rapid progression to cirrhosis and liver cancer in comparison with chronic hepatitis B (CHB) monoinfection.[1,2] Until recently, the only treatment option against CHD recommended by international guidelines was pegylated interferon-α2a (PegIFN).[3] Both as monotherapy and in combination with nucleos(t)ide analogues (NAs), PegIFN rarely achieved long-term HDV-RNA suppression, and tolerability issues have been reported frequently.[4,5] In summer 2020, the EMA conditionally approved the use of bulevirtide (BLV), a competitive inhibitor of the HBV/HDV entry receptor on hepatocytes, in patients with CHD.[6] This decision was made based on promising preliminary results of two phase II trials, in which BLV treatment was well tolerated and induced virologic response (*i.e.* a reduction of HDV-RNA by ≥2 $\log_{10}$ or to undetectable levels) and biochemical response (*i.e.* alanine aminotransferase [ALT] normalisation) in a majority of patients.[7] Since its conditional approval, only one clinical phase II study on BLV treatment has been fully published, confirming previous safety and efficacy data.[8] However, short-term treatment of 24 weeks of 2 mg/day BLV monotherapy led to HDV-RNA suppression below the lower

Keywords: Antivirals; Viral hepatitis; Cirrhosis; Hepatitis D; Treatment.
Received 28 February 2023; accepted 17 March 2023; available online 7 April 2023
† TR and PF share senior authorship.
‡ Author deceased.
* Corresponding author. Address: Division of Gastroenterology and Hepatology, Department of Medicine III, Medical University of Vienna, Vienna, Austria Gastroenterology Office 7i red, Vienna General Hospital, Waehringer Guertel 18-20, A1090, Vienna, Austria. Tel.: +4314040065890; Fax: +4314040047350 (M. Jachs) (T. Reiberger).
E-mail addresses: mathias.jachs@meduniwien.ac.at (M. Jachs), thomas.reiberger@meduniwien.ac.at (T. Reiberger).

limit of detection of the used assay in only 1 of 28 patients. Discontinuation of BLV monotherapy resulted in rapid rebound of HDV-RNA to pretreatment levels in most patients. Based on these data, no conclusions regarding the optimal treatment duration can be drawn. The label currently states that treatment should be continued as long as it is associated with clinical benefit.[9] Considering the rebounds in HDV-RNA after treatment discontinuation in patients who still had detectable HDV-RNA included in the phase II clinical study, stopping BLV treatment in patients who have not achieved complete HDV-RNA suppression seems counterintuitive. However, the rationale for incremental clinical benefit of continuous treatment in long-term suppressed patients remains to be established, considering that the drug must be prepared and s.c. injected daily by the patients, and anecdotical reports exist on hypersensitivity to the drug, which may limit the drug's long-term or even lifelong applicability in some patients.[10]

Therefore, clinical reasoning as well as patient preference and local expertise currently guide BLV treatment. In our recently published study, we tried to optimise treatment outcome using a response-guided BLV (±PegIFN) algorithm.[11] We suggested that treatment discontinuation may be considered for in patients who achieve long-term HDV suppression, that is, negative HDV-RNA levels. In this study, we report the outcomes of Austrian patients in whom BLV was discontinued following long-term HDV-RNA suppression.

Patients and methods

Patient cohort and study design

Six of the patients included in this report participated in the prospective Austrian BLV registry study that was previously published.[11] Briefly, the study included 23 patients who started BLV monotherapy (69.6% with cirrhosis). PegIFN was added in case of virologic non-response (*i.e.* a <2 log_{10} decline in HDV-RNA by Week 24) or if patients reached a virologic plateau (*i.e.* showed no further decreases in HDV-RNA after ≥24 weeks of BLV monotherapy). As of January 2023, 6 of 23 patients who were included in the Austrian BLV registry study, 5 under BLV monotherapy and 1 under combined BLV + PegIFN treatment, achieved treatment-induced negative HDV-RNA levels, corresponding to an overall HDV-RNA negativity rate of 31.6% in a per-protocol analysis. Thirteen patients are still HDV-RNA positive and on treatment. Two underwent transplantation, one permanently dropped out owing to incompliance, and one discontinued BLV and PegIFN treatment owing to adverse effects.

One additional patient, who was treated with a modified treatment regime (BLV + PegIFN initiated simultaneously), was included in this report. BLV (+ PegIFN in n = 2) treatment was stopped in all seven patients (Medical University of Vienna, n = 6; Medical University of Innsbruck, n = 1). After stopping treatment, patients were closely monitored at the discretion of the treating physician. Demographic, clinical, laboratory, and virologic parameters were collected. Follow-up was assessed until end of January 2023.

Laboratory tests and liver elastography

Routine laboratory tests were performed by the ISO-certified Department of Laboratory Medicine of the Medical University of Vienna or by the Department of Laboratory Medicine of the Medical University of Innsbruck.

HBeAg status was determined using commercially available chemiluminescent immunoassays. The quantitative assessment of HBsAg was conducted applying the Abbott ARCHITECT® assay (Abbott Diagnostics, Abbot Park, IL, USA) with a lower limit of linear quantification of 0.05 IU/ml.[12] As previously reported,[11] in Vienna, quantitative HDV-RNA levels were evaluated applying an HDV-RNA-PCR assay developed according to an external reference[13] with a lower limit of linear quantification of 100 copies/ml, whereas in Innsbruck, the RoboGene® HDV Quantification Kit 2.0 (Roboscreen Diagnostics, Leipzig, Germany) with a lower limit of linear quantification of 8 IU/ml was used. As shown previously, both assays show comparable sensitivity for the detection of HDV-RNA.[11] A head-to-head comparison of the two assays over the BLV treatment course of patient No. 1 is shown in Fig. S1. HDV-RNA levels assessed via the RoboGene® assay were converted to copies/ml by applying a conversion factor of 37, as previously described.[11] The HDV genotype was determined by Sanger sequencing of an approximately 1,000-bp PCR product for each sample (genome positions ~350–1,260, which includes most of the HDAg open reading frame), aligning them to representatives of the eight major genotypes,[14] and determining relatedness using a maximum likelihood tree with the GTR-G substitution model.

Assessment of liver fibrosis and stiffness

Liver biopsy was conducted in six of seven patients before BLV initiation. Liver stiffness before stopping BLV was assessed applying vibration-controlled transient elastography, that is, the FibroScan® system (EchoSense, Paris, France). Measurements were conducted according to international recommendations.[15]

Efficacy and safety parameters

Virologic, biochemical, and combined responses at BLV discontinuation were assessed according to the definitions used in clinical studies.[7] Safety was assessed mainly by monitoring ALT levels following the discontinuation of BLV and by clinical examination during regular clinical follow-up visits. ALT flares were defined as increases in ALT to a final value of 5 × upper limit of normal (ULN), that is, ≥200 U/L, according to the definition used in clinical trials.[8] In analogy to clinical studies assessing the off-treatment response to BLV, the sustainability of HDV suppression following BLV discontinuation was defined by undetectable HDV-RNA levels at follow-up week 24.[8]

Statistical analysis

Statistical analysis was conducted using GraphPad Prism 8 (GraphPad Software, La Jolla, CA, USA). Continuous parameters are shown as median (range), whereas categorical parameters are given as numbers (percentage) of patients with the respective attribute.

Ethics

All included patients gave informed written consent before inclusion in the prospective Austrian registry on BLV treatment, and both the parent study and this retrospective evaluation, which were conducted in accordance with the Declaration of Helsinki, have been approved by the institutional review board of the Medical University of Vienna (No. 1515/2020 and No. 2139/2021).

Results

Overall characteristics of the study population

Seven patients (four females and three males, age ranging from 31 to 68 years) were included in this report. Individual baseline characteristics referring to the time of treatment discontinuation

are summarised in Table 1. Four patients had cirrhosis, and two had already developed clinically significant portal hypertension. The treatment-naïve HDV-RNA levels ranged from 2.00 to 7.20 $\log_{10}$ copies/ml, and HDV genotype 1 was diagnosed in six of seven patients, whereas the HDV genotype was unknown in one patient. The duration of BLV treatment before discontinuation ranged from 46 to 141 weeks, and all patients achieved ≥24 weeks of undetectable HDV-RNA at the end of treatment, except for one patient whose treatment ended after only 12 weeks of suppression because social insurance did not reimburse further therapy. Except for one, all patients were on concomitant NA treatment and had suppressed HBV-DNA levels before, during, and at the end of BLV treatment, whereas quantitative HBsAg levels ranged from 3.08 to 4.37 $\log_{10}$ IU/ml at BLV cessation.

Patient No. 1

The patient was a 68-year-old woman born in Uzbekistan with compensated cirrhosis but no evidence for portal hypertension. Parts of her treatment history before and following BLV discontinuation have been previously published.[11,16] After almost 3 years of treatment and following 39 weeks of undetectable HDV-RNA, BLV treatment was stopped. HDV-RNA and ALT levels remained undetectable and normal, respectively, for ≥24 weeks of follow-up, as shown in Fig. 1. At Week 43 after cessation, HDV-RNA was detected again; however, ALT levels did not rise until Week 62 follow-up. Throughout the treatment-free period, the patient remained asymptomatic. Owing to the virologic and biochemical relapse, BLV 2 mg/day was reintroduced at Week 65. BLV was well tolerated again, and a similar response to the patient's first treatment was observed, as is evident from rapidly normalising ALT and decreasing HDV-RNA levels. So far, the patient has completed 46 weeks of retreatment, and HDV-RNA was undetectable at the last two recorded visits.

Patient No. 2

This patient was a 53-year-old man born in Turkey, who had compensated cirrhosis and subclinical portal hypertension evident from a hepatic venous pressure gradient of 7 mmHg.[17] The patient had completed 60 weeks of BLV monotherapy, achieving HDV-RNA suppression for a total of 12 weeks, but had to discontinue treatment before reaching 24 weeks of suppression. HDV-RNA was negative and ALT levels were normal at a follow-up visit at Week 3 after BLV discontinuation; however, HDV-RNA became detectable again at Week 13 (at 100 copies/ml). Owing to the patient's advanced disease, BLV was immediately reintroduced despite persistently normal ALT levels. Treatment was well tolerated. However, during the following 60 weeks of retreatment, HDV-RNA could not be sustainably suppressed again. Despite short intervals of 12 and 8 weeks of HDV-RNA negativity, HDV-RNA became repeatedly detectable again (always at 100 copies/ml). Transient noncompliance to daily injections of BLV after a total of almost 3 years of BLV treatment cannot be ruled out in this patient. Thus, PegIFN 180 µg/week was added. Twelve weeks later, HDV-RNA became undetectable, whereas ALT levels rose expectedly. The patient has completed 99 weeks of retreatment with BLV so far.

Patient No. 3

This 40-year-old woman from Mongolia with only low-grade fibrosis but pronounced treatment-naïve inflammatory activity had achieved undetectable HDV-RNA levels after only 27 weeks of BLV monotherapy. Following 24 weeks of HDV suppression, the treatment was discontinued. Already at the first follow-up visit 4 weeks later, HDV-RNA was detectable again; however, ALT levels (61 U/L) were only slightly elevated. Although HDV-RNA remained at 100 copies/ml, that is, the lower limit of detection, during the treatment-free follow-up, ALT levels rose to 160 U/ml (4 × ULN) by Week 14, as shown in Fig. 1. Therefore, BLV was reintroduced, resulting in a rapid ALT decline to normal levels in the following weeks. The patient reached HDV-RNA suppression again after 17 weeks of retreatment. So far, she has completed 30 weeks of retreatment.

Table 1. Patient and treatment characteristics of the patient cohort at BLV discontinuation.

Patient characteristics	P1	P2	P3	P4	P5	P6	P7
Sex (M/F)	F	M	F	F	M	M	F
Age (years)	68	53	40	31	53	54	37
Disease stage							
Fibrosis (METAVIR)	F4	F4	F1	F0/F1*	F2	F4	F4
Liver stiffness (kPa)	12.1	28.9	8.5	5.5	10.9	23.3	19
Cirrhosis (yes/no)	(+)	(+)	(-)	(-)	(-)	(+)	(+)
Portal hypertension (yes/no)	(-)	(+)	(-)	(-)	(-)	(-)	(+)
Treatment							
Duration of BLV treatment (weeks)	141	60	51	124	46	67	80
Duration of HDV-RNA suppression (weeks)	39	12	24	69	29	31	60
NA treatment (ETV/TDF)	TDF	ETV	TDF	TDF	ETV	TDF	ETV
IFN cotherapy (yes/no)	(-)	(-)	(-)	(+)	(+)	(-)	(-)
Virology							
HDV genotype	1	1	1	1	Unknown	1	1
HDV-RNA prior treatment ($\log_{10}$ copies/ml)	6.77	4.08	3.36	7.20	7.15	3.11	2.00
HBeAg (positive/negative)	(-)	(-)	(-)	(+)	(-)	(-)	(-)
HBsAg ($\log_{10}$ IU/ml)	4.03	3.22	3.56	4.37	4.05	3.09	3.08
Biochemistry							
Platelet count (G/L)	220	110	173	288	246	180	76
Bilirubin (mg/dl)	0.34	0.82	0.80	0.50	1.74	0.54	0.53
ALT (U/L)	22	31	33	214†	85	53	24
Albumin (g/dl)	35.8	48.8	47.0	38.8	45.7	45.8	48.7

ALT, alanine aminotransferase; BLV, bulevirtide; ETV, entecavir; F, female; IFN, interferon; M, male; P, patient; TDF, tenofovir disoproxile fumarate.
* No biopsy was performed in this patient. Fibrosis stage was estimated based on liver stiffness.
† The patient underwent continuous interferon treatment at BLV discontinuation.

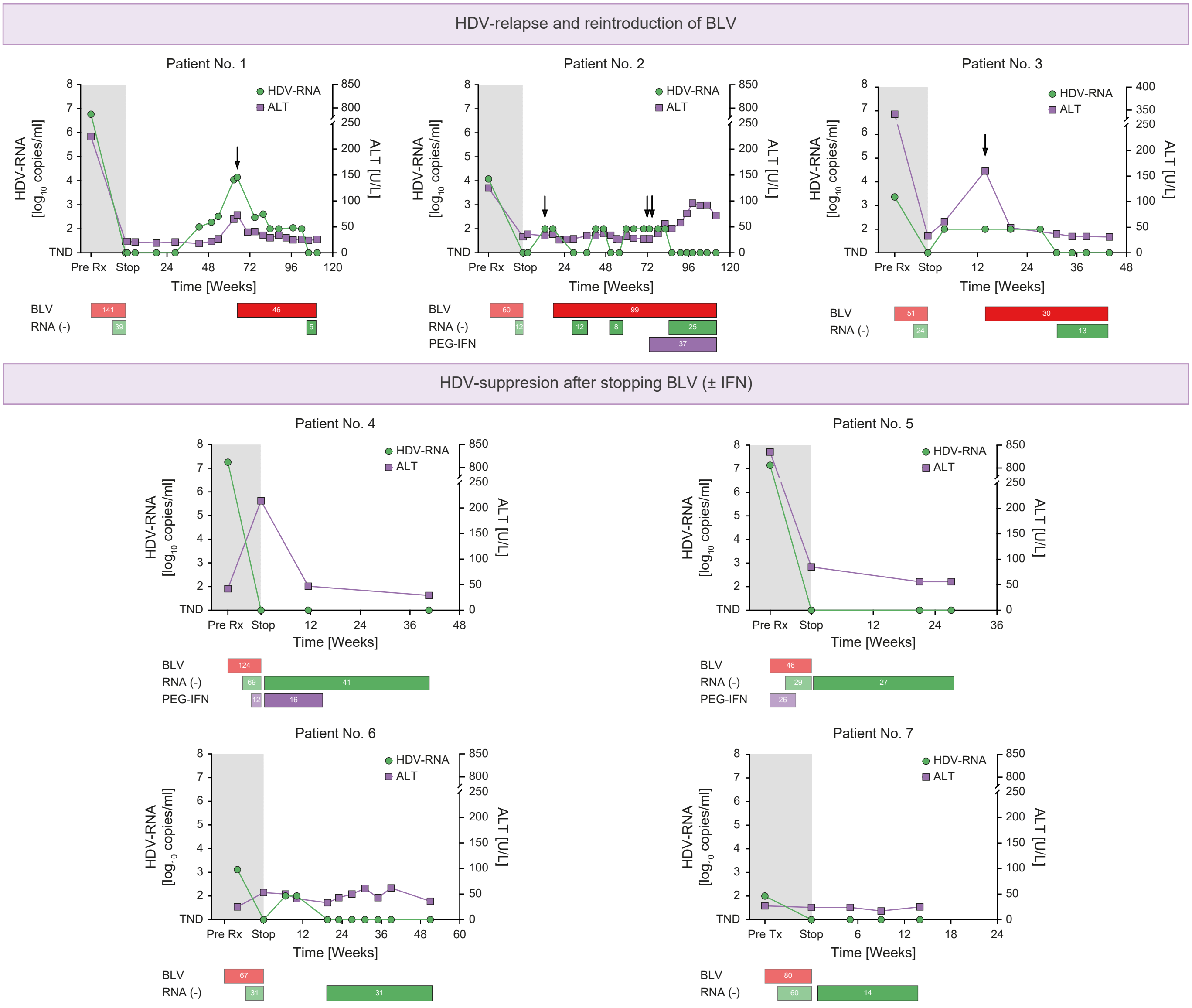

Fig. 1. HDV-RNA and ALT levels in six patients who reached 24 weeks of follow-up after BLV discontinuation, stratified by HDV relapse *vs.* HDV suppression at the last follow-up. BLV and IFN treatment and HDV-RNA suppression duration pre-/post-BLV cessation are indicated for each patient. BLV and IFN (re) introduction during follow-up is highlighted by one and two arrows, respectively. ALT, alanine aminotransferase; BLV, bulevirtide; IFN, interferon; PegIFN, pegylated interferon-α2a.

Patient No. 4

This 31-year-old Romanian woman with low-grade fibrosis had very high HDV-RNA and slightly elevated ALT levels before BLV treatment. The patient underwent two full years of BLV treatment and had undetectable HDV-RNA for a total of 69 weeks. BLV was discontinued at Week 124; however, owing to the patient's wish, PegIFN 90 µg/week was started 12 weeks before discontinuation, explaining the elevated ALT levels (214 U/L) at the end of BLV treatment. During follow-up, ALT levels declined despite ongoing PegIFN treatment and BLV cessation, and HDV-RNA remained undetectable. PegIFN was discontinued 16 weeks after BLV owing to tolerability issues. So far, the patient has completed 41 weeks of BLV-free and 25 weeks of overall treatment-free follow-up, and her ALT and HDV-RNA levels are normal and undetectable, respectively.

Patient No. 5

This 53-year-old male patient, who was born in Moldavia, was diagnosed with CHD in 1984. A liver biopsy in 1993 showed advanced fibrosis (F3). Following repeated unsuccessful treatments with standard interferon (IFN), a second liver biopsy in 1999 showed maintained high inflammatory activity (A3) and advanced fibrosis (F3). The patient received PegIFN from May to December 2003, resulting in ≥24 weeks of HDV-RNA undetectability by the method available at that time.[18] However, HDV-RNA was detected again a few years thereafter using a more sensitive assay. The last biopsy obtained in 2010 showed F2 fibrosis. HDV-RNA levels only slightly decreased during another cycle of 96 weeks PegIFN/entecavir between 2013 and 2014. The patient remained on entecavir, but infrequently attended follow-up visits. In mid-2019, the patient's ALT was at 81 U/L, HBV-DNA

was at very low levels, HDV-RNA was at 3.3 $\log_{10}$ copies/ml, and liver stiffness measurement showed F1. For unclear reasons, the patient stopped entecavir in March 2020. During a routine visit shortly thereafter, extraordinarily high ALT (835 U/L) and HBV-DNA (7.6 $\log_{10}$ IU/ml) and a marked increase in HDV-RNA to 7.1 $\log_{10}$ copies/ml were recorded. Fortunately, liver function was preserved, and thus, triple therapy with entecavir, BLV, and PegIFN (180 µg/week) was initiated shortly after. The changes in viral parameters on antiviral treatment is shown in Fig. 2. After 26 weeks of combined treatment, PegIFN had to be discontinued because of tolerability issues. Following a total of 46 weeks of BLV treatment and 29 weeks of HDV-RNA suppression, BLV was also discontinued. So far, the patient has completed 27 weeks of follow-up on entecavir monotherapy, during which no relapse in HDV-RNA was recorded, and a slight decrease in ALT was observed. Notably, HBV-DNA, for the first time during and after therapy, was negative at the last recorded visit, and quantitative HBsAg is steadily declining.

Patient No. 6

The patient was a 54-year-old Georgian native, who had compensated cirrhosis without portal hypertension. The patient underwent 67 weeks of BLV monotherapy, achieving HDV-RNA suppression for a period of 31 weeks. Notably, ALT levels increased slightly during therapy to a final value of 53 U/L, most likely related to the patient's pronounced metabolic comorbidity and obesity (BMI at BLV discontinuation, 39.5 kg/m^2). After stopping BLV, HDV-RNA was detectable again at the lower limit of quantification, that is, 100 copies/ml of the used highly sensitive assay, at Weeks 7 and 11; however, ALT even decreased

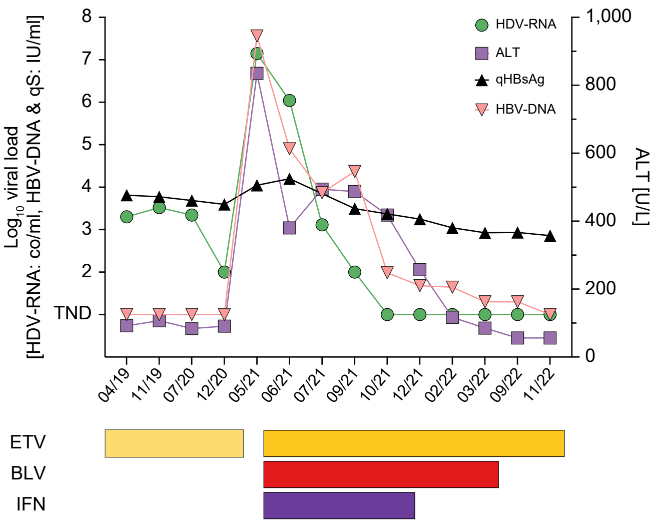

Fig. 2. Changes in virologic parameter in patient No. 5. Shortly after stopping ETV, marked increases of ALT (835 U/L), HBV-DNA (7.6 $\log_{10}$ IU/ml), and HDV-RNA (7.1 $\log_{10}$ copies/ml) were detected. Triple therapy with ETV, BLV, and PegIFN (180 µg/week) was initiated. ALT levels declined rapidly; HDV-RNA, for the first time ever using a highly sensitive assay, became rapidly undetectable after 17 weeks of treatment and remained undetectable for a total of 29 weeks. HBV-DNA, although still detectable during treatment, was swiftly suppressed. After 26 weeks, PegIFN was discontinued because of adverse effects, and 13 weeks later, BLV was stopped. So far, the patient has completed 27 weeks of follow-up on ETV monotherapy. HDV-RNA remains undetectable, and qHBsAg is continuously declining. ALT, alanine aminotransferase; BLV, bulevirtide; ETV, entecavir; IFN, interferon; PegIFN, pegylated interferon-α2a; qHBsAg, quantitative HBsAg.

slightly. Thus, BLV was not immediately reintroduced. Interestingly, HDV-RNA became undetectable again at Week 20 and remained negative for the remaining 31 weeks of follow-up.

Patient No. 7

This 37-year-old woman from Saudi Arabia had developed compensated cirrhosis with clinically significant portal hypertension before BLV treatment. HDV-RNA levels became undetectable at Week 20 of BLV treatment and remained negative for further 60 weeks of therapy. ALT levels were normal before and throughout BLV treatment. The patient has only recently stopped BLV treatment; however, following the discontinuation of BLV, ALT levels remained within normal ranges, and HDV-RNA was still suppressed at Week 14 of follow-up.

Overall safety and efficacy analysis

Despite increases in ALT in two patients, all patients remained asymptomatic after stopping BLV. No clinically significant events were recorded during a follow-up period of 14 to 112 weeks.

Week 24 of BLV-free follow-up has been reached by six of seven patients included in this study. Among those, three (50%) showed a relapse before Week 24, whereas one additional relapse occurred after 43 weeks. Notably, in one patient who showed an early relapse, HDV-RNA could be again detected for only 4 weeks shortly after BLV withdrawal, and HDV-RNA became undetectable thereafter again. Meanwhile, two patients who were treated with BLV + PegIFN maintained suppressed HDV-RNA levels during ≥24 weeks of follow-up. Furthermore, as shown in Fig. 3, HBsAg levels remained stable in all patients after stopping treatment. A detailed description of HBsAg levels at treatment initiation, treatment withdrawal, and the last recorded follow-up visit is given in Table S1 for each patient. BLV was reintroduced in three patients, and all achieved virologic response again, when considering the treatment-naïve HDV-RNA levels as the comparator/baseline value. Bile acid levels were monitored in Viennese (n = 6) patients. Although treatment induced an expected increase in bile acids, they decreased to normal levels following the discontinuation of BLV in all patients. Retreatment was paralleled by a resurgence of bile acid levels to elevated ranges, as shown in Fig. 3.

Discussion

This report shows for the first time, long-term follow-up data of patients in whom HDV-RNA became undetectable on treatment with a BLV-containing regimen. BLV discontinuation was offered to seven patients included in the prospective Austrian BLV cohort study achieving long-term HDV-RNA suppression with or without concomitant PegIFN therapy.[11] All patients remained on (continuous) NA treatment. Among the six patients who completed 24 weeks of BLV-free follow-up, early relapses occurred in three patients, and one additional late relapse was observed after almost 1 year. No early HDV relapses were seen in the two patients who underwent concomitant PegIFN treatment. No safety signals were recorded, and no ALT flares were observed within short-term follow-up, regardless of the underlying disease severity. This contrasts the high rate of ALT flares that were observed in approximately 25% of unsuppressed patients who discontinued BLV 2 mg/day as part of the MYR202 study.[8]

The course of patient No. 5 is quite unusual. Initially, HBV and HDV infection were well controlled on long-term entecavir therapy. For unclear reasons, the patient stopped entecavir, and

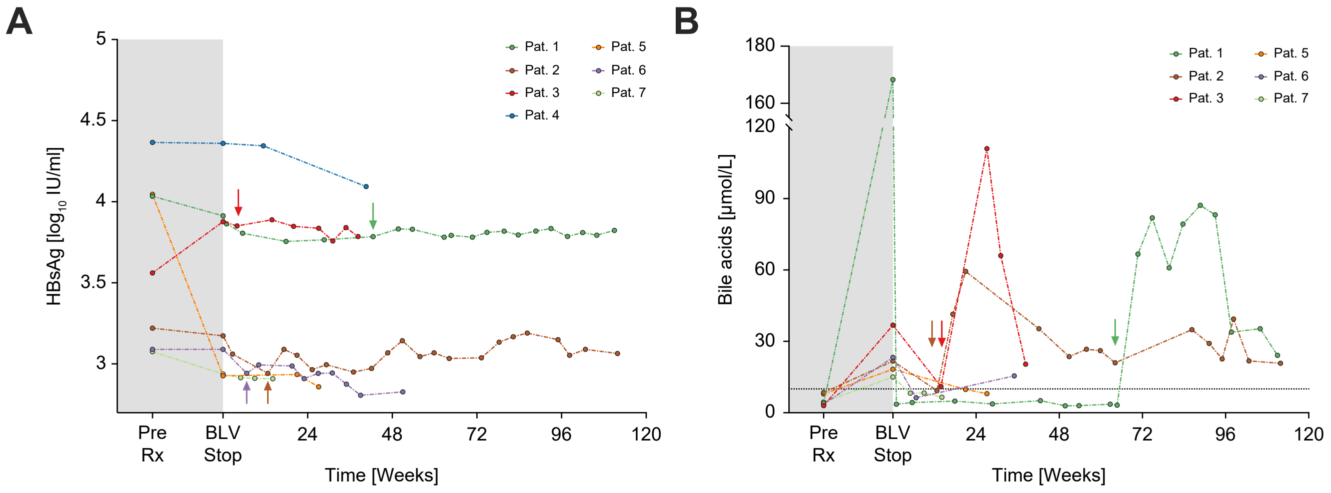

Fig. 3. Quantitative HBsAg levels and bile acid levels before treatment, at BLV discontinuation, and during follow-up in the overall cohort. HDV relapses (A) and BLV reintroductions (B) are indicated by arrows in the patient's corresponding colour. The horizontal line in (B) indicated the upper limit of normal of the used bile acid assay, which was 10 μmol/L. BLV, bulevirtide.

shortly thereafter, both virus levels and ALT increased sharply. Thus, in contrast to the other six patients, he had a very high HBV viral load before BLV + PegIFN treatment. Triple therapy led to a rapid viral suppression in our patient, and HDV-RNA became negative and remained undetectable during follow-up. This resembles previously reported approaches of immunologic priming provided by a short course of prednisone followed by rapid removal that enabled effective treatment with IFN in selected patients with CHB.[19,20] Although stopping NA may induce HBsAg loss in some patients, its safety is not well studied, and response rates may vary according to age, genotype, and HBsAg levels. In a recent study, stopping NA induced ALT flares to a final value of >10 × ULN in 31% and to >5 × ULN in another 16% of patients.[21] No patients experienced hepatic decompensation or died. In another study, 33 of 166 patients switching from NA to PegIFN experienced viral breakthrough.[22] Importantly, all studies investigating stopping NA in CHB excluded patients with HDV coinfection. Along these lines, stopping NA therapy should be carefully scrutinised in CHD responding to anti-HDV treatment, as suppression of HDV may induce an increase in HBV concentrations, which could, in turn, promote ALT flares.[23]

Our observations in a small number of well-characterised patients do not solve the question how long BLV therapy should be continued in patients with sustained HDV-RNA suppression on treatment. Similar to NA treatment in CHB, when to stop BLV in patients who show virologic response without achieving HDV-RNA undetectability is unknown. Meanwhile, evidence for the incremental clinical value of continuous or indefinite treatment in patients who achieve HDV-RNA negativity is lacking.

An important aspect to be studied is the necessity and timing of concomitant, immunostimulatory PegIFN therapy.[24] The reported synergistic short-term effects of combined therapy with BLV and PegIFN[25] might not be needed to achieve virologic response in the long run, as recently demonstrated in an update on the non-randomised French early access programme, where similar response rates were reported in patients undergoing BLV monotherapy or combined BLV and PegIFN after 2 years of treatment.[26] However, PegIFN might facilitate the induction of sustainable HDV suppression following long-term BLV treatment, enabling long treatment-free intervals in patients with CHD. Moreover, it should be noted that PegIFN, in contrast to BLV treatment, has an effect on HBV as well as HDV, and, ultimately, sustainable long-term suppression or 'cure' of CHD seems unlikely without concomitant 'functional cure' of CHB, that is, HBsAg loss.[27] Along these lines, it should be noted that different (surrogate) endpoints for the evaluation of the efficacy of emergent drugs targeted at CHD are currently being investigated in clinical trials.[28] However, all investigated endpoints need to prove their prognostic values for liver-related outcomes, and the optimal way to evaluate treatment success remains unknown as of today. Importantly, these issues also apply to (surrogate) endpoints for off-treatment efficacy.

Ultimately, three patients who relapsed at any point during follow-up restarted BLV treatment. All patients tolerated the therapy well, and all responded to retreatment. PegIFN was added in one patient to achieve sustained suppression of HDV-RNA during retreatment. Of note, when considering treatment-naïve HDV-RNA levels as a comparator, the patient had previously achieved virologic response during retreatment, as is evident from a ≥2 $\log_{10}$ decline in viral load. Overall, the desirable target of year-long drug-free intervals in most patients with CHD treated with BLV seems elusive as of today. Stopping BLV led to rapid relapses within <24 weeks in almost all patients in the MYR 202 trial,[8] and late relapses during ≥24 weeks of BLV-free follow-up seem likely, as evidenced not only by the one late relapse recorded within our cohort but also by the high rate of late relapses after PegIFN monotherapy.[29] However, no conclusion regarding late relapses can be drawn from our study, as most patients did not have ≥48 of follow-up data available. Thus, continuous close monitoring of patients opting to stop BLV is warranted until further data emerge. Based on our observations and pharmacodynamic considerations,[6] it appears unlikely that stopping BLV adversely influences the efficacy of a second cycle of therapy using the same drug. In turn, the efficacy and safety of long-term treatment of BLV need to be further studied, most importantly with regard to long-term elevations of bile acids.

Our retrospective study has some limitations that clearly suggest the need for further studies. First, in the absence of guidelines,

the treatment regimen, duration, and exact stopping moment was not standardised, resulting in different durations of HDV-RNA suppression and time of follow-up within our cohort that was treated outside a clinical trial setting. Second, the seven patients were quite heterogeneous with respect to the stage of chronic liver disease, spanning from mild fibrosis to advanced compensated cirrhosis with pronounced clinically significant portal hypertension. Thus, no stage-specific conclusions regarding efficacy and safety of BLV cessation can be derived from our cohort. Along these lines, PegIFN was added in one patient who did not achieve sustainable HDV-RNA suppression upon BLV retreatment. This patient had very advanced compensated cirrhosis with portal hypertension. Generally, PegIFN is safe in patients with compensated advanced chronic liver disease but should only be applied by clinicians with expertise in IFN therapy. This 'rescue' option might be unavailable for patients with very advanced disease. Third, in the absence of guidance from earlier studies, the proposed stopping rule of ≥24 weeks of HDV-RNA negativity was arbitrarily chosen, and the optimal on-treatment efficacy endpoint indicating a safe and effective opportunity to withdraw BLV treatment remains to be defined. Considering the high rate of HDV-RNA relapses in the BLV monotherapy group within our cohort, even longer intervals of HDV-RNA suppression should be investigated in future studies. The sensitivity of the assay used to assess HDV-RNA levels should be considered in this regard. In our case, highly and similarly sensitive assays were used. Thus, our results may not be translatable to settings where assays with a lower sensitivity threshold are applied. Generally, there is an unmet need to standardise HDV-RNA assays.[30] There are many tests commercially available, and usually, the locally available tests are chosen. Comparisons between the various tests have not yet been systemically conducted. In six of seven patients included in this report, a test developed according to Le Gal *et al.*[13] was applied. We previously demonstrated a similar sensitivity of the test applied in Vienna and the RoboGene® 2.0 assay, which were simultaneously used and compared head-to-head in patient No. 1 of this report.[11,16] This test was also used in a previous report by Asselah *et al.*[31] and is applied, among other tests, in the French ATU (EurobioPlex HDV® kit, Eurobio Scientific, Les Ulis, France). Overall, both assays show comparable sensitivity for the detection of HDV-RNA. Commercially available tests should be compared head-to-head. Finally, the decision to discontinue BLV treatment should be made on a case-by-case basis, factoring in all the mentioned caveats as well as patient's preference, as evidence for the risks and benefits and of indefinite BLV treatment remains be gathered in future collaborative efforts.

In conclusion, we demonstrate that stopping BLV treatment under close monitoring appears to be safe in a 'real-world' cohort of patients across all stages of chronic liver disease, although larger cohort studies – especially in patients with advanced disease – investigating the risks of BLV discontinuation are needed. Stopping BLV treatment might enable considerably long drug-free intervals without daily injections, particularly in patients who were treated with BLV and PegIFN combined. However, the optimal time point for stopping BLV treatment and patient selection remain to be investigated in future studies. Importantly, in patients with HDV-RNA relapse following discontinuation, BLV can be safely reintroduced, and antiviral efficacy was seemingly not impaired by previous exposure to the drug.

Abbreviations

ALT, alanine aminotransferase; BLV, bulevirtide; CHB, chronic hepatitis B; CHD, chronic hepatitis D; ETV, entecavir; IFN, interferon; NA, nucleos(t)ide analogue; PegIFN, pegylated interferon-α2a; TDF, tenofovir disoproxile fumarate; ULN, upper limit of normal.

Financial support

No funding specific to this study was received.

Conflicts of interest

MJ has served as a speaker for Gilead. PM served as a speaker and/or consultant and/or advisory board member for MSD, AbbVie, Intercept, and Gilead, and received travel support from Gilead and AbbVie. MM served as a speaker and/or consultant and/or advisory board member for AbbVie, Collective Acumen, Gilead, Takeda, and W. L. Gore & Associates, and received travel support from AbbVie and Gilead. MT served as a speaker and/or consultant and/or advisory board member for Abbvie, Albireo, BiomX, Boehringer Ingelheim, Bristol-Myers Squibb, Falk, Genfit, Gilead, Hightide, Intercept, Janssen, MSD, Novartis, Phenex, Regulus, Siemens, and Shire, and received travel support from AbbVie, Falk, Gilead, Intercept, and Jannsen, as well as grants/research support from Albireo, Alnylam, Cymabay, Falk, Gilead, Intercept, MSD, Takeda, and Ultragenyx. Moreover, he is a co-inventor of patents on the medical use of 24-norursodeoxycholic acid. HZ received speaker honoria from the Abbvie, Bayer, BMS, Falk Foundation, Gilead, Intercept, Merck, MSD, Novartis, Pierre-Fabre, Pharmacosmos, and Vifor; he has advised for Abbvie, Bayer, Eisai, Gilead, Intercept, MSD, Novartis, Novo Nordisk, Shire, Pierre-Fabre, Pharmacosmos, and Vifor. He further received travel grants from Abbvie, Bayer, Gilead, and Intercept, and research grants from Abbvie, Gilead, MSD, Novartis, Pharmacosmos, and Vifor. TR served as a speaker and/or consultant and/or advisory board member for AbbVie, Bayer, Boehringer Ingelheim, Gilead, Intercept, MSD, Siemens, and W. L. Gore & Associates, and received grants/research support from AbbVie, Boehringer Ingelheim, Gilead, MSD, Philips, and W. L. Gore & Associates, as well as travel support from Boehringer Ingelheim and Gilead. PF received an unrestricted research grant from Gilead, was a member of the safety review Committee for MyrPharma, received speaking honoraria from Gilead and Abbvie, and received a consulting/advisory board fee from Vivaraxx. MP, LH, MSch, and LB have nothing to disclose.

Please refer to the accompanying ICMJE disclosure forms for further details.

Authors' contributions

Conceptualisation: MJ, PF; Data curation: all authors; Formal analysis and visualisation: MJ; Writing of the original draft: MJ, PF; Reviewing and editing of the manuscript: all authors; Supervision: TR.

Data availability statement

Data will be made available upon reasonable request to the corresponding author.

Supplementary data

Supplementary data to this article can be found online at https://doi.org/10.1016/j.jhepr.2023.100751.

References

Author names in bold designate shared co-first authorship

[1] Miao Z, Zhang S, Ou X, Li S, Ma Z, Wang W, et al. Estimating the global prevalence, disease progression, and clinical outcome of hepatitis delta virus infection. J Infect Dis 2020;221:1677–1687.

[2] Jachs M, Binter T, Schmidbauer C, Hartl L, Strasser M, Laferl H, et al. Hepatitis D virus (HDV) prevalence in Austria is low but causes considerable morbidity due to fast progression to cirrhosis. United Eur Gastroenterol J 2021;9:1119–1127.

[3] European Association for the Study of the Liver. EASL 2017 Clinical Practice Guidelines on the management of hepatitis B virus infection. J Hepatol 2017;67:370–398.
[4] Wedemeyer H, Yurdaydìn C, Dalekos GN, Erhardt A, Çakaloğlu Y, Değertekin H, et al. Peginterferon plus adefovir versus either drug alone for hepatitis delta. N Engl J Med 2011;364:322–331.
[5] **Wedemeyer H, Yurdaydin C**, Hardtke S, Caruntu FA, Curescu MG, Yalcin K, et al. Peginterferon alfa-2a plus tenofovir disoproxil fumarate for hepatitis D (HIDIT-II): a randomised, placebo controlled, phase 2 trial. Lancet Infect Dis 2019;19:275–286.
[6] Urban S, Neumann-Haefelin C, Lampertico P. Hepatitis D virus in 2021: virology, immunology and new treatment approaches for a difficult-to-treat disease. Gut 2021;70:1782–1794.
[7] Lampertico P, Roulot D, Wedemeyer H. Bulevirtide with or without pegIFNα for patients with compensated chronic hepatitis delta: from clinical trials to real-world studies. J Hepatol 2022;77:1422–1430.
[8] Wedemeyer H, Schöneweis K, Bogomolov P, Blank A, Voronkova N, Stepanova T, et al. Safety and efficacy of bulevirtide in combination with tenofovir disoproxil fumarate in patients with hepatitis B virus and hepatitis D virus coinfection (MYR202): a multicentre, randomised, parallel-group, open-label, phase 2 trial. Lancet Infect Dis 2022;23:117–129.
[9] European Medicines Agency. Hepcludex – summary of product characteristics https://www.ema.europa.eu/en/medicines/human/EPAR/hepcludex. Accessed 03 December 2022.
[10] Schwarz C, Chromy D, Bangert C, Schwarz M, Jachs M, Reiberger T, et al. Immediate-type hypersensitivity reaction to bulevirtide and successful desensitization in a patient with HBV/HDV-associated compensated cirrhosis. J Hepatol 2022;77:254–255.
[11] Jachs M, Schwarz C, Panzer M, Binter T, Aberle SW, Hartl L, et al. Response-guided long-term treatment of chronic hepatitis D patients with bulevirtide-results of a “real world” study. Aliment Pharmacol Ther 2022;56:144–154.
[12] Strassl R, Reiberger T, Honsig C, Payer BA, Mandorfer M, Grabmeier-Pfistershammer K, et al. Viral determinants predicting hepatitis B surface antigen (HBsAg) seroclearance in HIV-/HBV-coinfected patients. J Viral Hepat 2014;21:508–516.
[13] Le Gal F, Gordien E, Affolabi D, Hanslik T, Alloui C, Dény P, et al. Quantification of hepatitis delta virus RNA in serum by consensus real-time PCR indicates different patterns of virological response to interferon therapy in chronically infected patients. J Clin Microbiol 2005;43:2363–2369.
[14] Le Gal F, Brichler S, Drugan T, Alloui C, Roulot D, Pawlotsky JM, et al. Genetic diversity and worldwide distribution of the deltavirus genus: a study of 2,152 clinical strains. Hepatology 2017;66:1826–1841.
[15] European Association for the Study of the Liver. EASL Clinical Practice Guidelines on non-invasive tests for evaluation of liver disease severity and prognosis – 2021 update. J Hepatol 2021;75:659–689.
[16] Loglio A, Ferenci P, Uceda Renteria SC, Tham CYL, Scholtes C, Holzmann H, et al. Safety and effectiveness of up to 3 years' bulevirtide monotherapy in patients with HDV-related cirrhosis. J Hepatol 2022;76:464–469.
[17] Reiberger T, Schwabl P, Trauner M, Peck-Radosavljevic M, Mandorfer M. Measurement of the hepatic venous pressure gradient and transjugular liver biopsy. J Vis Exp 2020. https://doi.org/10.3791/58819.
[18] Ferenci P, Formann E, Romeo R. Successful treatment of chronic hepatitis D with a short course of peginterferon alfa-2a. Am J Gastroenterol 2005;100:1626–1627.
[19] Perrillo RP, Regenstein FG, Peters MG, DeSchryver-Kecskemeti K, Bodicky CJ, Campbell CR, et al. Prednisone withdrawal followed by recombinant alpha interferon in the treatment of chronic type B hepatitis. A randomized, controlled trial. Ann Intern Med 1988;109:95–100.
[20] Perrillo RP, Schiff ER, Davis GL, Bodenheimer Jr HC, Lindsay K, Payne J, et al. A randomized, controlled trial of interferon alfa-2b alone and after prednisone withdrawal for the treatment of chronic hepatitis B. The Hepatitis Interventional Therapy Group. N Engl J Med 1990;323:295–301.
[21] Liem KS, Fung S, Wong DK, Yim C, Noureldin S, Chen J, et al. Limited sustained response after stopping nucleos(t)ide analogues in patients with chronic hepatitis B: results from a randomised controlled trial (Toronto STOP study). Gut 2019;68:2206–2213.
[22] Huang D, Yan W, Han M, Yuan W, Wang P, Chen Y, et al. Insufficient immunity led to virologic breakthrough in NAs-treated chronic hepatitis B patients switching to Peg-IFN-α. Antivir Res 2022;197:105220.
[23] Yurdaydin C, Keskin O, Kalkan Ç, Karakaya F, Çalişkan A, Karatayli E, et al. Optimizing lonafarnib treatment for the management of chronic delta hepatitis: the LOWR HDV-1 study. Hepatology 2018;67:1224–1236.
[24] Sandmann L, Wedemeyer H. Interferon-based treatment of chronic hepatitis. D Liver Int 2022. https://doi.org/10.1111/liv.15410.
[25] Zhang Z, Ni Y, Lempp FA, Walter L, Mutz P, Bartenschlager R, et al. Hepatitis D virus-induced interferon response and administered interferons control cell division-mediated virus spread. J Hepatol 2022;77:957–966.
[26] De Lédinghen V, Hermabessière P, Metivier S, Bardou J, Hilleret M, Loustaud-Ratti V, et al. Bulevirtide, with or without peg-interferon, in HDV infected patients in a real-life setting. Two-year results from the French multicenter early access program. Hepatology 2022;76:S26–S28.
[27] Ferenci P, Reiberger T, Jachs M. Treatment of chronic hepatitis D with bulevirtide – a fight against two foes – an update. Cells 2022;11:3531.
[28] Da BL. Clinical trials in hepatitis D virus: measuring success. Hepatology 2023;77:2147–2157.
[29] **Bremer B, Anastasiou OE**, Hardtke S, Caruntu FA, Curescu MG, Yalcin K, et al. Residual low HDV viraemia is associated HDV RNA relapse after PegIFNa-based antiviral treatment of hepatitis delta: results from the HIDIT-II study. Liver Int 2021;41:295–299.
[30] Stelzl E, Ciesek S, Cornberg M, Maasoumy B, Heim A, Chudy M, et al. Reliable quantification of plasma HDV RNA is of paramount importance for treatment monitoring: a European multicenter study. J Clin Virol 2021;142:104932.
[31] Asselah T, Loureiro D, Le Gal F, Narguet S, Brichler S, Bouton V, et al. Early virological response in six patients with hepatitis D virus infection and compensated cirrhosis treated with Bulevirtide in real-life. Liver Int 2021;41:1509–1517.

Journal of Hepatology, Volume *5*

Supplemental information

Long-term follow-up of patients discontinuing bulevirtide treatment upon long-term HDV-RNA suppression

Mathias Jachs, Marlene Panzer, Lukas Hartl, Michael Schwarz, Lorenz Balcar, Jeremy V. Camp, Petra Munda, Mattias Mandorfer, Michael Trauner, Stephan W. Aberle, Heinz Zoller, Thomas Reiberger, and Peter Ferenci

Supplementary material

Long-term follow up of patients discontinuing bulevirtide treatment upon long-term HDV-RNA suppression

Mathias Jachs, Marlene Panzer, Lukas Hartl, Michael Schwarz, Lorenz Balcar, Jeremy V. Camp, Petra Munda, Mattias Mandorfer, Michael Trauner, Stephan W. Aberle, Heinz Zoller, Thomas Reiberger, Peter Ferenci

Table of contents

Fig. S1 Page 2
Table S1 Page 3
References Page 4

Supplementary Figure legends

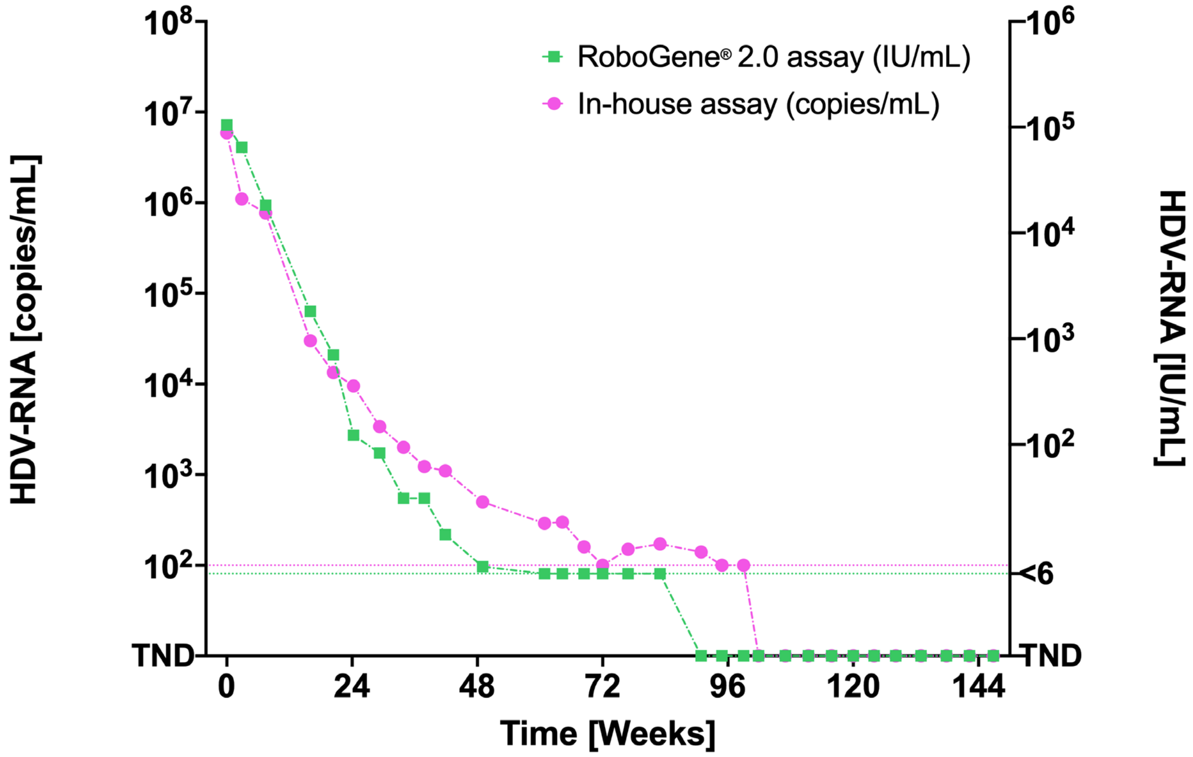

Fig. S1. Head-to-head comparison of the diagnostic performance of the HDV-RNA assay developed in-house according to Le Gal et al. [1] (lower limit of linear quantification: 100 copies/mL) used in six out of seven included patients, and the RoboGene ® 2.0 assay (lower limit of linear quantification: 6 IU/mL), over the bulevirtide (BLV) treatment course of patient #1. The assessment applying the RoboGene ® 2.0 assay was conducted externally [2].

Table S1

Patient No.	Treatment	qHBsAg (log_{10} IU/mL)		
		Treatment-naïve	BLV discontinuation	Last Follow-Up
**P1**	BLV	4.03	3.91	3.79
**P2**	BLV	3.22	3.17	3.06
**P3**	BLV	3.56	3.88	3.79
**P4**	BLV + PEG-IFN	4.37	4.36	4.09
**P5**	BLV + PEG-IFN	4.05	2.93	2.86
**P6**	BLV	3.09	3.09	2.88
**P7**	BLV	3.08	2.94	2.91

Table S1: Individual quantitative HBsAg (qHBsAg) levels at treatment initiation, BLV (± PEG-IFN) discontinuation, and at the last recorded follow-up visit.

References

[1] Le Gal F, Gordien E, Affolabi D, Hanslik T, Alloui C, Dény P, et al. Quantification of hepatitis delta virus RNA in serum by consensus real-time PCR indicates different patterns of virological response to interferon therapy in chronically infected patients. J Clin Microbiol 2005;43:2363-2369.

[2] Loglio A, Ferenci P, Uceda Renteria SC, Tham CYL, Scholtes C, Holzmann H, et al. Safety and effectiveness of up to 3 years' bulevirtide monotherapy in patients with HDV-related cirrhosis. J Hepatol 2022;76:464-469.
